# Integrated machine learning identifies disulfidptosis-related and ferroptosis-related genes to evaluate survival prognosis and treatment efficacy in kidney renal clear cell carcinoma

**DOI:** 10.1016/j.bbrep.2025.102102

**Published:** 2025-07-12

**Authors:** Yuan Xiang, Zijian Zhou, Tong Mu, Shunyao Zhang, Lei Xie, Yajie Zhou, Wenxiong Zhang, Liuxiang Fu

**Affiliations:** a Department of Thoracic Surgery, The Second Affiliated Hospital, Jiangxi Medical College, Nanchang University, Nanchang, 330006, China; b The Second Clinical Medical School, Jiangxi Medical College, Nanchang University, Nanchang, 330088, China; c Department of Urology, The Second Affiliated Hospital, Jiangxi Medical College, Nanchang University, Nanchang, 330006, China; d Emergency Department, The Second Affiliated Hospital, Jiangxi Medical College, Nanchang University, Nanchang, 330006, China

**Keywords:** Disulfidptosis, Ferroptosis, KIRC, DRFs, Prognostic signature, Drug sensitivity

## Abstract

**Background:**

Ferroptosis and disulfidptosis, two programmed cell death pathways, critically drive tumor growth by affecting metastasis. Although the prognostic value of disulfidptosis and ferroptosis had been separately validated in kidney renal clear cell carcinoma (KIRC), prognostic effect of integrating two programmed death genes remains unclear in KIRC. Our objective is to establish an innovative prognostic model for KIRC.

**Methods:**

We sourced KIRC patients’ information that contains clinical and genomic from The Cancer Genome Atlas (TCGA) database. We selected disulfidptosis-related and ferroptosis-related genes (DRFs) to construct a prognostic model. By combining clinical features and prognostic models, we developed the nomogram. Additionally, the mechanism of DRF was explored in KIRC, including tumor immune dysfunction and exclusion (TIDE), Kaplan-Meier (K-M) analysis, tumor microenvironment (TME) analysis, and more. Drug sensitivity analysis shows which drugs are sensitive to tumors. Experiment with RT-PCR to confirm DRFs gene expression in the cell line.

**Results:**

Constructing risk score with five DRFs, all tumor samples were categorized into high-risk group (HG) and low-risk group (LG). The HG samples demonstrated lower survival rates according to K-M survival curves. The nomogram with risk score demonstrated significant predictive value than nomogram without the risk score. TME analysis indicated that the proportion of T cells follicular helper and Tregs was higher in HG, while Macrophages M1 and Mast cells resting were higher in LG. GSEA analysis demonstrated Retinol metabolism pathway, drug metabolism other enzymes pathway, etc. were enriched in HG, while endocytosis-related pathway, neurotrophin signaling pathway, etc. were enriched in LG. TIDE analysis showed tumors in HG are more prone to immune evasion. The drug sensitivity analysis indicated that the HG is sensitive to antitumor drugs such as Cedrane and Osimertinib, while the LG is sensitive to antitumor drugs such as 5-Fluorouracil and Entinostat. RT-qPCR have confirmed expression of DRFs in KIRC cell lines.

**Conclusions:**

Our DRFs-based prognostic model and nomogram effectively predict survival and guide treatment decisions.

## Highlights

• First disulfidptosis/ferroptosis integrated prognostic model for KIRC.
• 5-gene signature and nomogram optimize survival prediction.
• Risk stratification reveals TMB-immune interplay and divergent drug sensitivity.

## 1 Introduction

As the most common renal parenchymal neoplasm, Clear cell renal cell carcinoma (ccRCC) demonstrates distinct molecular profiles including VHL gene mutations and aberrant HIF pathway activation, with incidence rates 2–3 times higher than papillary and chromophobe subtypes combined [1]. According to NCCN 2023 Guidelines (Version 2), the TNM classification framework stratifies ccRCC into four progressive stages (I-IV), with corresponding 5-year survival rates ranging from 93 % (Stage I) to 8 % (Stage IV) as documented in the SEER-22 registry [2]. However, since many kidney cancer cases are detected incidentally, prognosis predictions based on clinical staging alone are often unreliable [3]. This underscores the need for a more accurate diagnostic and prognostic approach. Recently, advances in molecular mechanism research have led to the development of biomarker-based prognostic models, which show great promise in predicting cancer patient outcomes.

Disulfidptosis involves redox imbalance (NADPH depletion and disulfide stress), while ferroptosis features iron-dependent lipid peroxidation, both contributing to tumorigenic microenvironments through differential metabolic disruptions [4,5]. The combined model of ferroptosis and disulfidptosis developed by Ma et al. has been validated for prognostic assessment in lung adenocarcinoma patients [6]. A similar prognostic model developed by Zhang C et al. has been validated for prognosis in patients with hepatocellular cells [7]. Zhang X et al. reviewed the contribution of DRFs in the treatment of bladder cancer [8]. However, we found that no one has used DRFs to construct a prognostic model of KIRC.

Consequently, we used DRFs to developed a prognostic model. The clinical utility of this model was evaluated using enrichment analysis, drug sensitivity analysis, TME analysis, TIDE analysis and TMB analysis.

## 2 Methods

### 2.1 Source of data

The original cohort data were derived from TCGA database (https://portal.gdc.cancer.gov/repository), which included transcriptome sequencing data of 537 patients and clinical annotation information (survival time, stage, grade, etc.) of 614 patients. We used transcription data in HTSeq-Counts and HTSeq-FPKM formats, each serving different analytical purposes. We selected patients with comprehensive transcriptomic and clinical data, ensuring they were present in both datasets. Consequently, this study included 529 cancer samples and 86 normal samples. For independent verification of the analytical model's generalizability, the research team employed the publicly accessible E-MTAB-1980 genomic dataset, which was obtained through the ArrayExpress (https://www.ebi.ac.uk/arrayexpress/) - a well-established bioinformatics platform maintained by the European Bioinformatics Institute for disseminating functional genomics experiments.

Ferroptosis-associated genes (n = 383) and disulfidptosis regulators (n = 29) were systematically identified using FerrDb (http://www.zhounan.org/ferrdb/current/) and literature-based screening. These genes were selected based on their established roles in ferroptosis and disulfidptosis, which are critical processes in tumor progression [9,10].

### 2.2 Select DRFs genes

DRFs were assessed using Pearson's correlation with stringent significance thresholds (|r| > 0.4, p < 0.001). This cohort was subsequently partitioned via stratified random sampling into derivation and validation subsets at a 1:1 ratio. Within the derivation cohort, prognostic feature selection employed a three-stage analytical pipeline: (1) preliminary screening through univariate Cox proportional hazards regression; (2) dimensional reduction via LASSO (Least Absolute Shrinkage and Selection Operator); (3) multivariate Cox modeling to identify independent prognostic determinants, controlling for potential confounders.

### 2.3 KIRC DRFs prognostic model

Model Development and Risk Stratification. The model was constructed using risk scores derived from the formula:

Risk score = $\sum_{i=1}^{n}\left(\text{coefficient}\left({\text{gene}}_{i}\right)\times \text{expression}\left({\text{gene}}_{i}\right)\right)$.

A midpoint risk threshold (median-derived) was used to classify patients into HG and LG. To assess survival outcomes, OS differences across training, validation, and pooled datasets were evaluated with the "survival" package in R. For genomic profiling, principal component analysis (PCA) dimensionality reduction was implemented across four distinct genomic groupings: pan-transcriptome genes, signature-defined prognostic genes, disulfidptosis-associated markers, and ferroptosis pathway regulators, thereby validating intergroup heterogeneity. Finally, survival outcomes in the HG and LG groups were examined across diverse clinical subgroups (e.g., age, stage) to identify factors influencing prognosis.

### 2.4 Nomogram development for patient survival prediction

Using the "RMS" package in R software, we constructed a prognostic nomogram that integrates key clinical factors, including age, risk group, and tumor stage. This nomogram serves as a comprehensive and practical tool to predict patient outcomes, providing a visual representation of the combined impact of these clinical variables on survival. By quantifying the contribution of each factor, the nomogram offers a personalized approach for assessing prognosis in clinical settings [11]. Using the " survival " and " survminer "package in R software, we constructed Progression-Free Survival (PFS).

### 2.5 Enrichment analysis

Under the model constructed with 5 DRFs, we categorized the cases into HG and LG. We used the GSEA_4.3.2 software [12] to filter pathways with a p-value of <0.05 and a false discovery rate (FDR) of <0.25. Using the 5 DRFs, patients were classified into HG and LG. Pathway enrichment was conducted with GSEA against the Hallmark gene sets, identifying significantly dysregulated pathways (nominal *P* < 0.05, FDR<0.25). Additionally, we applied KEGG enrichment assays and GESA enrichment assays to investigate potential functions and pathways that correlate with gene sets.

### 2.6 Tumor mutational burden and prognostic assessment

TMB is a crucial factor in evaluating tumor prognosis. We retrieved somatic mutation data with the "TCGAbiolinks" package. Somatic variant analysis was performed using the maftools package (v2.6.05) with mutation annotation format (MAF) files processed through a standardized bioinformatic pipeline, and calculated the corresponding TMB values [13,14]. Additionally, we further explored the survival outcomes of patients under different mutation conditions to reflect the impact of various mutations on patient prognosis.

### 2.7 Immune microenvironment profiling

ESTIMATE scores were computed using the “ESTIMATE” package, followed by comparative analysis between HG and LG. To enhance immune landscape characterization, additional tumor microenvironment data were sourced from TIMER 2.0 platform (http://timer.cistrome.org) [15]. Immune ecosystem profiling through an integrated multi-algorithm framework (CIBERSORT, xCell, etc.) demonstrated significant risk score-immunome correlations, validated by deconvolution concordance analysis across all seven platforms. Finally, immune infiltration dynamics were quantified across 22 functionally distinct cell types to comprehensively map shifts in immune cell proportions.

### 2.8 Comparing the DRFs in immunotherapy and chemotherapy

To explore tumor Immunotherapy across risk groups, TIDE scores were acquired from the database (http://tide.dfci.harvard.edu/) [16]. GSVA was executed using the R package (v1.46.0) to systematically characterize immune microenvironment heterogeneity and delineate risk-stratified immunological fingerprints. Drug Sensitivity Prediction. Antimicrobial susceptibility profiling was conducted to pinpoint chemotherapeutic agents with differential efficacy between risk groups. Therapeutic responsiveness was quantified through estimation of half-maximal inhibitory potency (IC50) for common anticancer agents via the "oncoPredict" algorithm [17], leveraging transcriptomic profiles to predict drug efficacy. Comparative analysis of these metrics across HG and LG subgroups facilitated risk-adapted treatment planning.

### 2.9 Validation by RT-qPCR

Total RNA isolation from KIRC cell line models was conducted employing TRIzol reagent (Life Technologies, CA, USA), with subsequent random sampling for quantitative reverse transcription PCR validation. The amplification reactions were executed on the Applied Biosystems 7500 Fast platform using BlazeTaq SYBR Green qPCR master mix (GeneCopoeia, Guangzhou, China), following standardized thermal cycling protocols. Each RNA sample was evaluated in three independent experiments. For the study, the HK2 cell line served as the normal control, while 769-P and 786-O represented the tumor cell lines. Primer sequences for the DRFs are provided in Table S1, and their relative expression levels were determined using the 2ˆ(-ΔΔCt) method.

We examined the protein expression of DRFs by comparing KIRC tumor tissues with normal tissues. Data for this comparison was accessed from the HPA database (https://www.proteinatlas.org/).

## 3 Results

### 3.1 Selection of DRFs signature in KIRC patients

**Fig. 1:**
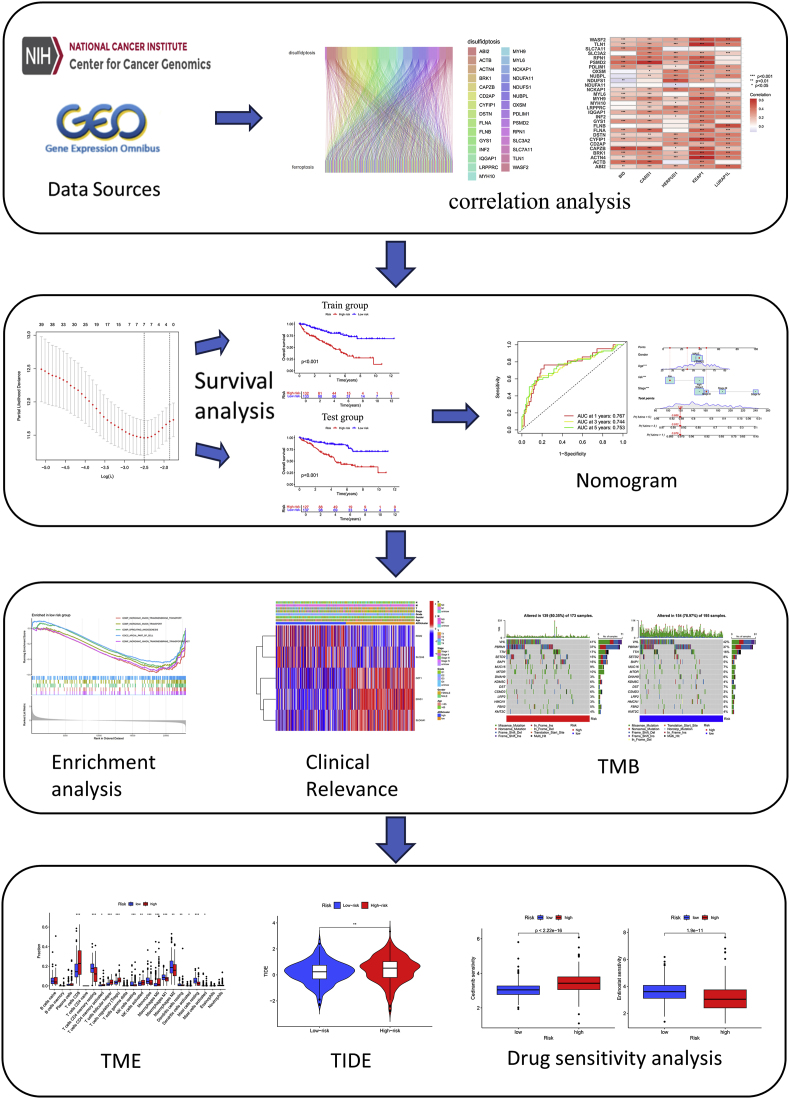
Flowchart of the entire study design.

**Fig. 2:**
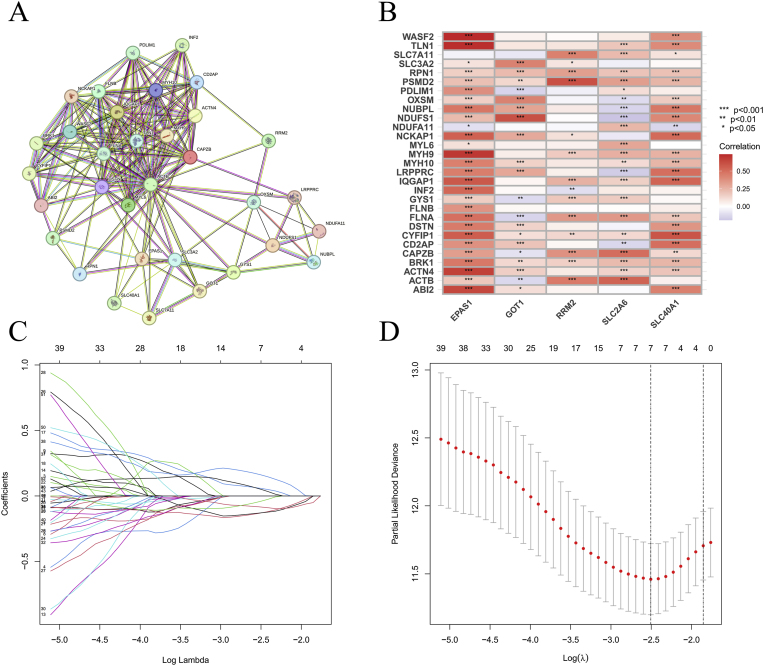
Screening for DRFs. PPI network of DRFs (A); Correlation between ferroptosis-related genes and disulfidptosis genes (B); Genes were screened using the LASSO algorithm (*C*–D).

**Fig. S1:**
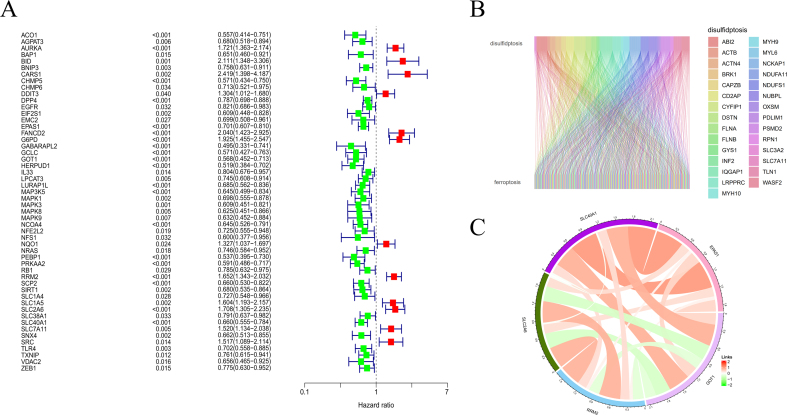
Constructing a prognostic model. Forest plot related to univariate analysis (A); correlation diagram of ferroptosis-related genes and disulfidptosis genes (B); chord diagram showing the correlations among 5 DRFs.

The framework is summarized in Fig. 1. Pearson correlation analysis identified 341 DRFs (Fig. 2B), followed by the generation of protein-protein interaction (PPI) network to map functional relationships among these DRFs (Fig. 2A). patients’ information is detailed in Table 1. Through univariate Cox regression, 52 DRFs were further screened as prognostically significant (Table S2). The hazard ratio of these 52 DRFs are displayed in Fig. S1A. Subsequent LASSO analysis revealed 8 DRFs (Fig. 2C–D), and we further refined our selection using multivariate Cox regression analysis, ultimately identifying 5 of the most significant DRFs (Table S3). Subsequently, we examined the correlation of these 5 DRFs (Fig. S1C).

**Table 1:** Clinical information of the patients in the test and training groups.

Characteristics	Train cohort (n = 265)		Test cohort (n = 264)		Entire cohort (n = 529)	
	n	%	n	%	n	%
Age						
≤65	172	64.91	176	66.67	348	65.78
>65	93	35.09	88	33.33	181	34.22
**Status**						
Alive	174	65.66	182	68.94	356	67.3
Dead	91	34.34	82	31.06	173	32.7
**Gender**						
Female	94	35.47	91	34.47	185	34.97
Male	171	64.53	173	65.53	344	65.03
**Stage**						
Stage I	130	49.06	133	50.38	263	49.72
Stage II	28	10.57	29	10.98	57	10.78
Stage III	60	22.64	63	23.86	123	23.25
Stage IV	46	17.35	37	14.02	83	15.68
Unknow	1	0.38	2	0.76	3	0.57
**T stage**						
T1	133	50.19	136	51.52	269	50.85
T2	34	12.83	35	13.26	69	13.04
T3	92	34.72	88	33.33	180	34.03
T4	6	2.26	5	1.89	11	2.08
**M stage**						
M0	202	76.23	218	82.58	420	79.4
M1	46	17.36	33	12.5	79	14.93
Unknow	17	6.41	13	4.92	30	5.67
**N stage**						
N0	116	43.77	123	46.59	239	45.18
N1	4	1.51	12	4.55	16	3.02
Unknow	145	54.72	129	48.86	274	51.8
**Race**						
White	229	86.42	231	87.5	460	86.96
Black or African American	28	10.57	26	9.85	54	10.21
Asian	5	1.89	3	1.14	8	1.51
Unknow	3	1.12	4	1.51	7	1.32

**Abbreviations:** T stage: Tumor stage; N stage: Node stage; M stage: metastasis stage.

### 3.2 Building and validating the prognostic model

**Fig. 3:**
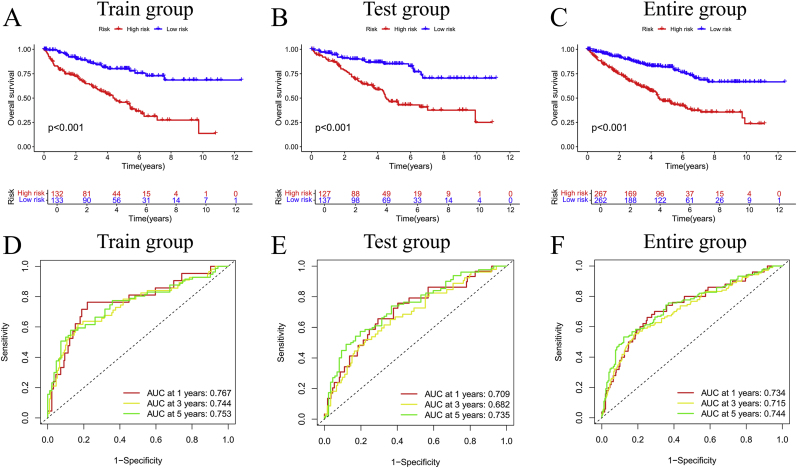
The prognostic effect was validated in three groups. Survival curves in high-risk and low-risk groups (A–C) and ROC between high-risk and low-risk groups (D–F).

Using the expression levels of five DRFs, we computed individual risk scores. Risk score = coefficient (EPAS1) × expression (EPAS1) + coefficient (GOT1) × expression (GOT1) + coefficient (RRM2) × expression (RRM2) + coefficient (SLC2A6) × expression (SLC2A6) + coefficient (SLC40A1) × expression (SLC40A1). Table 2 presents the patient information for both the HG and LG. We tested the survival rates of the HG and LG across different cohorts. High-risk patients showed markedly worse survival versus low-risk group with robust predictive accuracy (Fig. 3A–C).

**Table 2:** Clinical information for 529 patients in different risk categories.

Characteristics	High-risk group (n = 267)		Low-risk group (n = 262)	
	n	%	n	%
Age				
≤65	172	64.42	176	67.18
>65	95	35.58	86	32.82
**Status**				
Alive	141	52.81	215	82.06
Dead	126	47.19	47	17.94
**Gender**				
Female	88	32.96	97	37.02
Male	179	67.04	165	62.98
**Stage**				
Stage I	96	35.96	167	63.74
Stage II	28	10.49	29	11.07
Stage III	81	30.34	42	16.03
Stage IV	60	22.46	23	8.78
Unknow	2	0.75	1	0.38
**T stage**				
T1	99	37.08	170	64.89
T2	37	13.86	32	12.21
T3	122	45.69	58	22.14
T4	9	3.37	2	0.76
**M stage**				
M0	196	73.41	224	85.5
M1	57	21.35	22	8.4
Unknow	14	5.24	16	6.1
**N stage**				
N0	130	48.69	109	41.5
N1	13	4.87	3	1.15
Unknow	124	46.44	150	57.25
**Race**				
White	235	88.01	225	85.88
Black or African American	25	9.36	29	11.07
Asian	4	1.5	4	1.53
Unknow	3	1.13	4	1.52

**Abbreviations:** T stage: Tumor stage; N stage: Node stage; M stage: metastasis stage.

**Fig. S2:**
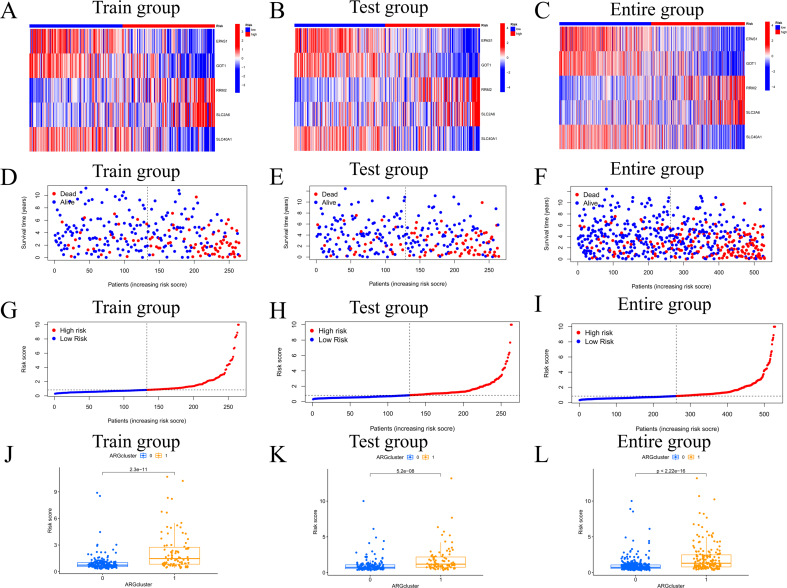
The predictive performance of the model was validated using the training group, testing group, and the entire cohort. Heatmaps showing the expression of 5 DRFs (A-C). Risk scores representing each patient's survival status (D-F) and scatter plots of risk curves (G-I). Scatter plots of risk scores for patients with different survival statuses (J-L).

Time-dependent ROC analysis demonstrated robust prognostic performance across all cohorts. In the training group, the model achieved AUC values of 0.767 (1-year), 0.744 (3-year), and 0.753 (5-year). Comparable predictive accuracy was observed in the test group, with 1-year, 3-year, and 5-year AUCs of 0.709, 0.682, and 0.735, respectively. When analyzed as a combined cohort, the integrated AUCs remained stable at 0.734 (1-year), 0.715 (3-year), and 0.744 (5-year), underscoring the model generalizability (Fig. 3D–F). Additionally, various visualizations-including DRFs expression profiles (Fig. S2A–C), risk curves (Fig. S2D–F), risk distribution maps (Fig. S2G–I), and scatter plots (Fig. S2J–L)-The combinatorial signature demonstrated robust prognostic value across validation phases.

**Fig. 4:**
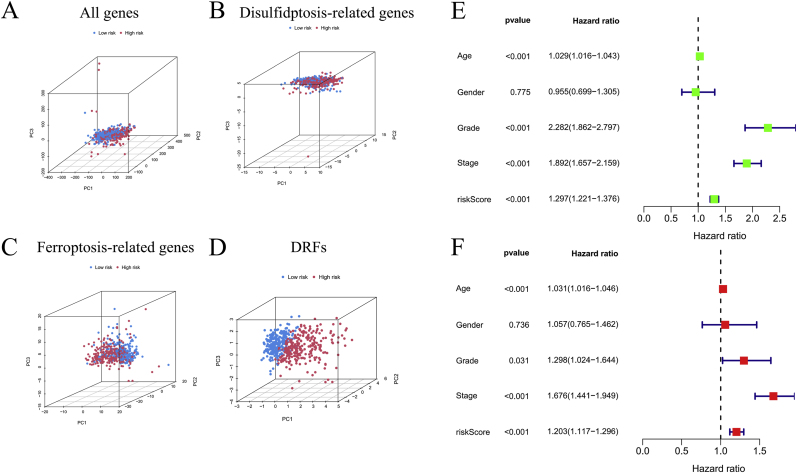
PCA and independent prognostic analysis of biomarkers. PCA based on all genes (A), disulfidptosis-related genes (B), ferroptosis-related genes (C), and DRFs signature (D); Univariate (E) and multivariate (F) independent prognostic analyses.

Univariable cox regression identified four independent factors with statistical significance: age (HR = 1.08, P = 0.008), disease stage (HR = 2.34, P < 0.001), risk score (HR = 1.92, P = 0.002), and tumor grade (HR = 1.55, P = 0.019). Intriguingly, gender showed no significant association with survival outcomes (P = 0.432) (Fig. 4E). Importantly, multivariable analysis confirmed that age (HR = 1.05, P = 0.038), disease stage (HR = 2.17, P = 0.003), and risk score (HR = 1.76, P = 0.011) retained their statistical significance after adjusting for confounding variables (Fig. 4F).

**Fig. 5:**
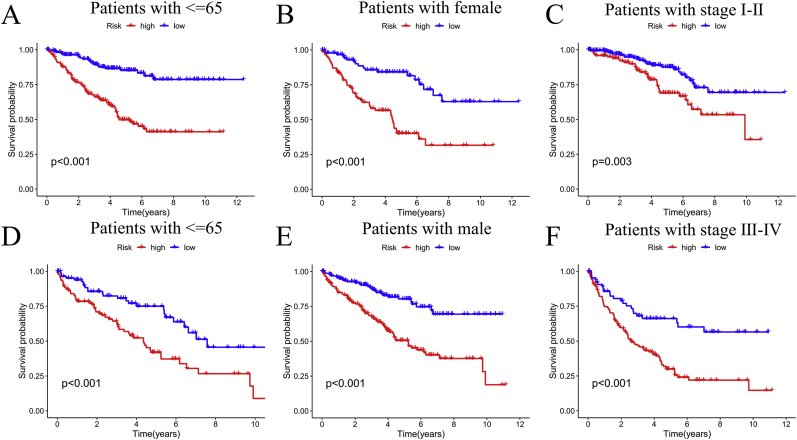
Model validation under other clinical features. Survival analysis based on different clinical characteristics (A–F).

**Fig. S3:**
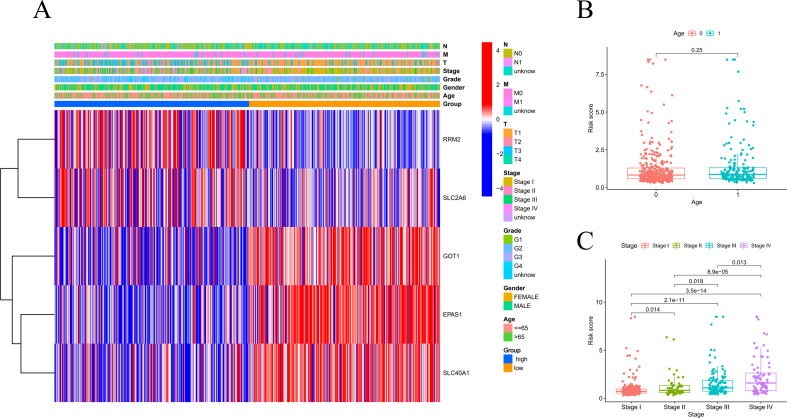
Validation of the model's effectiveness. Heatmap of DRFs feature expression with clinical information (A); correlation analysis of risk features with age (B) and stage (C).

The heatmap related to the clinical data and risk group (Fig. S3A), as well as the survival curves of patients under various clinical conditions (Fig. 5), demonstrate the importance of predictive ability. PFS analysis demonstrated significantly prolonged progression-free survival in the low-risk group compared to the high-risk cohort (Fig. S11).

### 3.3 Overall survival prediction nomogram

**Fig. 6:**
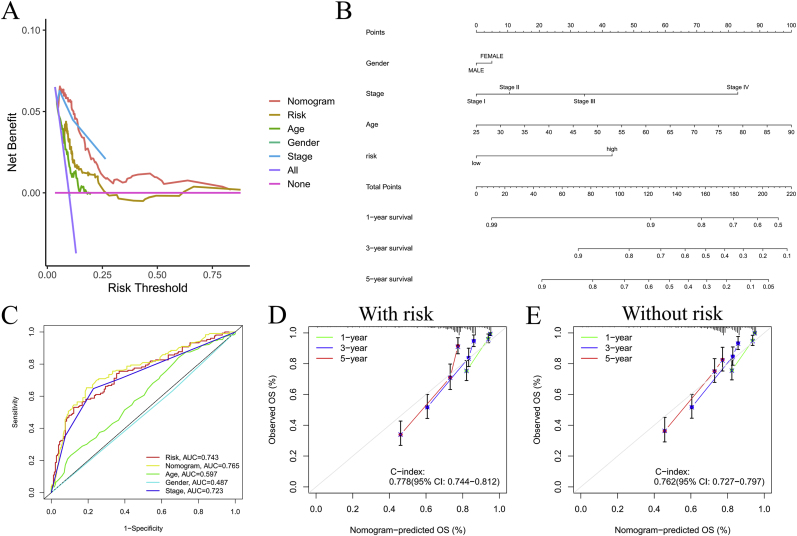
Build a nomogram. Decision curve analysis across different clinical characteristics (A); A nomogram that combines gender, age, period, and risk (B); ROC curves for various clinical features (C); Nomograms for patients with and without risk factors (D–E).

**Fig. S4:**
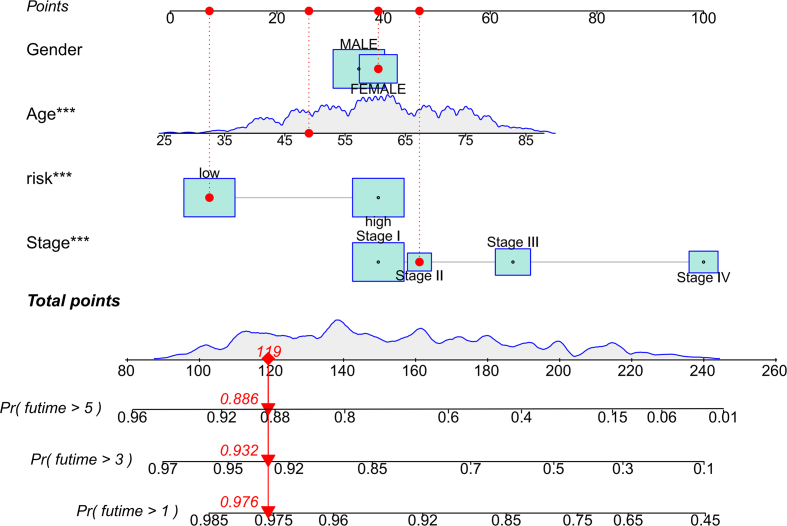
Nomogram with patient verification.

A scatterplot (Fig. S3C) demonstrated the correlation between tumor stage and risk score. Although age appears to contribute to an increased risk score, its association was not statistically significant (P = 0.25) (Fig. S3B). Both DCA (Fig. 6A) curve and ROC showed good predictive ability of the model. Risk AUC, nomogram AUC, age AUC, gender AUC, and stage AUC in the ROC curve were 0.743, 0.765, 0.597, 0.487, and 0.723, respectively (Fig. 6C). Additionally, we developed a risk estimator for tumor patients incorporating age, risk score, gender, and tumor stage (Fig. 6B), which was validated with patient data and showed robust performance (Fig. S4). The nomogram with risk score (Fig. 6D) demonstrated significant predictive value than nomogram without the risk score (Fig. 6E).

### 3.4 KEGG and GSEA enrichment analysis

**Fig. S5:**
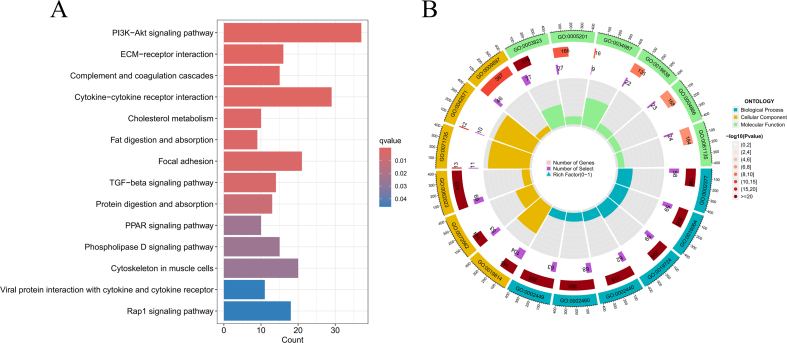
Enrichment analysis. Bar chart of KEGG enrichment analysis (A); pie chart of GO analysis (B).

**Fig. S6:**
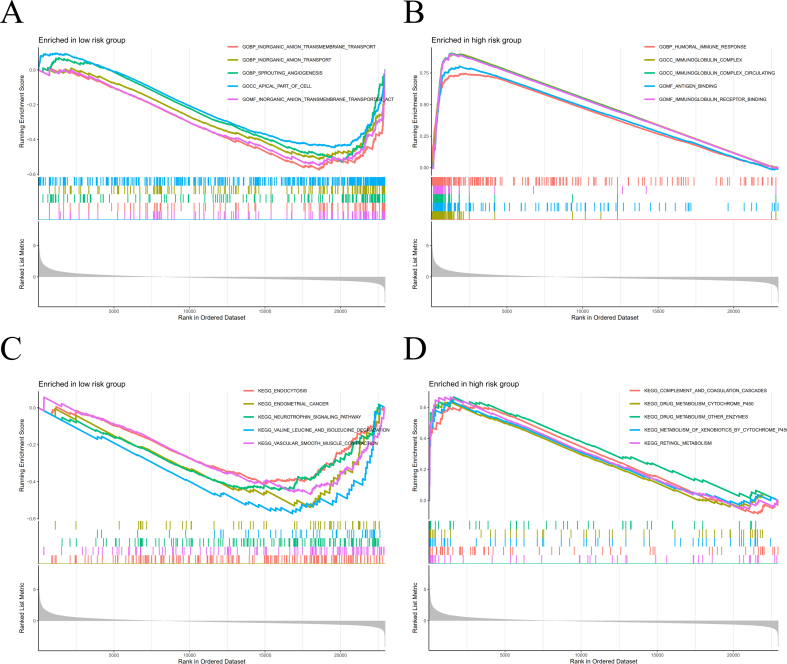
GSEA enrichment analysis. Enriched pathways in low-risk populations across different gene sets (A, C); enriched pathways in high-risk populations across different gene sets (B, D).

We use KEGG analysis to revealed that DRFs are linked to several key pathways, including cytokine–cytokine receptor interactions, the TGF–beta pathway, the PI3K–Akt pathway, coagulation cascades, and interactions between viral proteins and cytokine receptors (Fig. S5). Meanwhile, GSEA indicated that various gene sets are enriched in different pathways. Specifically, in the HG, KEGG analysis showed important enrichment in pathways such as cytokine receptor interactions, complement and coagulation cascades, cytochrome P450-mediated drug metabolism, and lupus erythematosus (Fig. S6). Detailed pathway information is provided in Table S4.

#### 3.4.1 TMB analysis

**Fig. 7:**
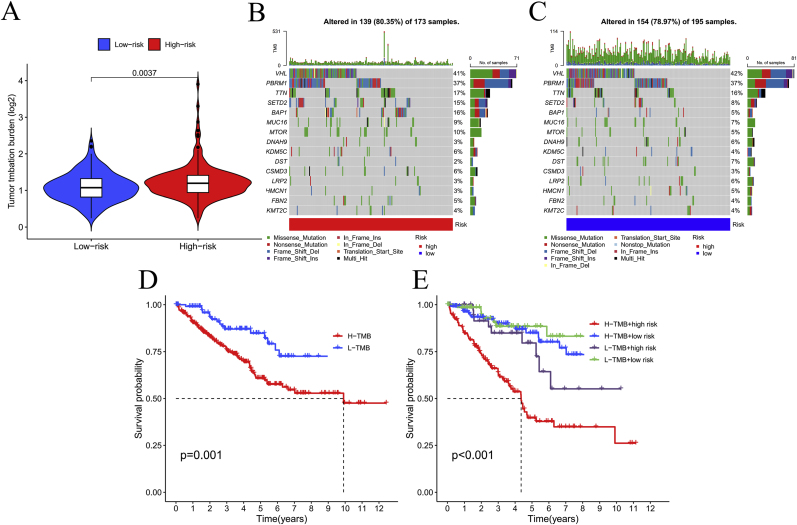
Tumor mutation burden across different risk groups. Mutational burden in different groups (A); Mutations in the high-risk group (B); Mutations in the low-risk group (C); Survival analysis of varying mutation burdens in high and low-risk groups (D); Survival analysis of different mutation groups (E).

Mutational Landscape and Survival Implications. TMB was elevated in the HG compared to the LG (P = 0.0037) (Fig. 7A). Analysis of the top 15 mutated genes across risk subgroups (Fig. 7B–C) revealed distinct mutational profiles: HG: SETD2 (15 %), VHL (41 %), TTN (17 %), PBRM1 (37 %) and BAP1 (16 %).LG: VHL (42 %), PBRM1 (37 %), TTN (16 %), SETD2 (8 %), and MUC16 (7 %).Kaplan-Meier survival curves stratified by mutational burden (Fig. 7D–E) demonstrated that elevated TMB correlated with reduced survival outcomes (P < 0.05).

#### 3.4.2 TME analysis

**Fig. 8:**
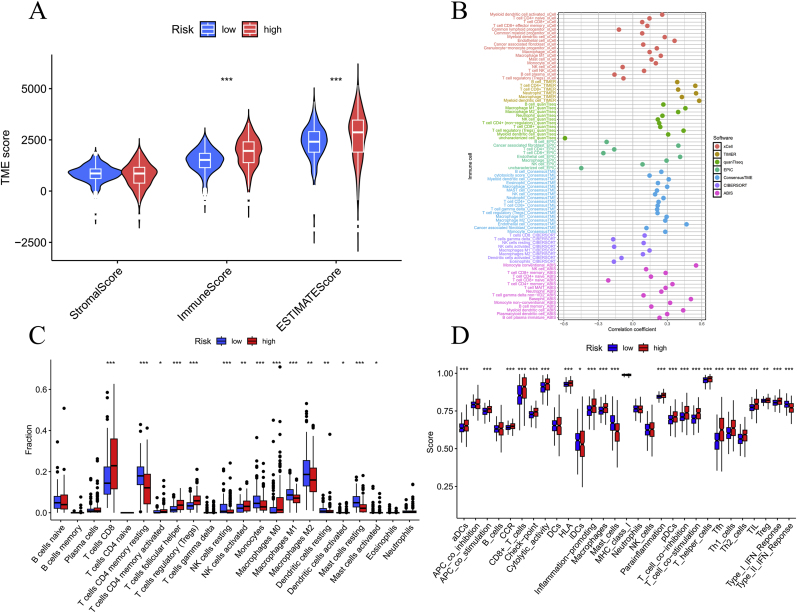
For the analysis of the immune microenvironment. Violin plots showing the differences in immune scores, stromal scores, and ESTIMATE scores among different risk subgroups (A); the correlation between immune cells and risk scores (B); the proportions of 22 immune cell types in the two subgroups as determined by the CIBERSORT algorithm (C); single-sample gene set enrichment analysis (D). ∗p < 0.05, ∗∗p < 0.01, ∗∗∗p < 0.001.

**Fig. S7:**
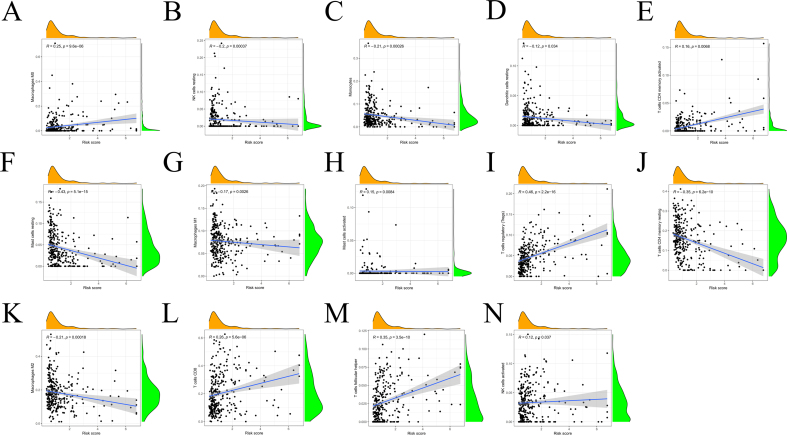
Correlation plot between risk scores and immune cells. Scatter plot of the correlation between risk scores and Macrophages M0 (A), NK cells resting (B), Monocytes (C), Dendritic cells resting (D), T cells CD4 memory activated (E), Mast cells resting (F), Macrophages M1 (G), Mast cells activated (H), T cells regulatory (I), T cells CD4 memory resting (J), Macrophages M2 (K), T cells CD8 (L), T cells follicular helper (M), NK cells activated (N).

We calculated three immunization scores using TME analysis and found that the HG scored higher than the LG in both the immune score and the estimate core and was statistically significant (Fig. 8A). Multi-algorithm deconvolution revealed significant risk score-immunocyte correlations (Fig. 8B). Distinct immune microenvironment patterns were observed between risk stratifications (Fig. 8C). The high-risk cohort demonstrated significant enrichment of cytotoxic (CD8^+^ T cells, P < 0.05) and pro-inflammatory lymphocyte subsets, including activated CD4^+^ memory T cells (P < 0.01), follicular helper T cells (P < 0.001), along with immunosuppressive components (Tregs, P < 0.001; M0 macrophages, P < 0.001). In comparison, LG showed predominant infiltration of immune surveillance effectors: resting CD4^+^ memory T cells (P < 0.001) and NK cells (P < 0.01), complemented by anti-inflammatory myeloid populations (M1 macrophages and monocytes, both P < 0.01) alongside quiescent dendritic (P < 0.01). Fig. S7 illustrates the associations among immune cell profiles and prognostic risk stratification.

High-risk KIRC patients exhibited a functionally paradoxical immune microenvironment characterized by concurrent activation of antitumor mechanisms and adaptive immunosuppression (Fig. 8D). These cases demonstrated enhanced antigen presentation through activated dendritic cells (aDCs, P < 0.001) and amplified APC co-stimulation (P < 0.05), coupled with elevated cytolytic activity (P < 0.001) and CCR-mediated chemotaxis (P < 0.001). Paradoxically, this pro-inflammatory state - marked by heightened HLA expression (P < 0.05), type I IFN responses (P < 0.01), and Th1/Th2/Tfh cell infiltration (P < 0.001) - coexisted with T cell exhaustion signatures, evidenced by co-elevated co-stimulatory/inhibitory checkpoints (P < 0.001). Simultaneously, critical immunoregulatory functions were suppressed, including impaired iDC maturation (P < 0.01), attenuated type II IFN signaling (P < 0.001), and mast cell dysfunction (P < 0.001). This dual activation/suppression pattern suggests evolved immune evasion through macrophage-driven chronic inflammation (IL-6/IL-1β axis) and PD-1/STAT1-mediated TIL exhaustion, while compromised antiviral responses facilitate tumor immunoediting.

### 3.5 Benefits of model in the treatment of KIRC

**Fig. S8:**
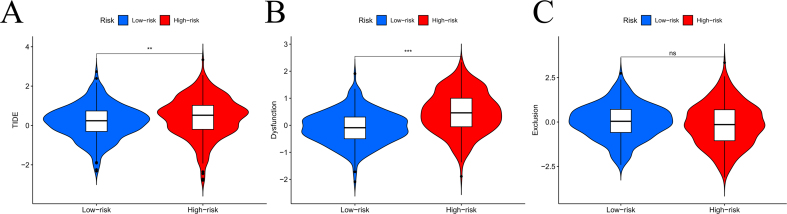
Risk patterns of DRFs in cancer treatment. Differences in TIDE (A), dysfunction (B), and immune exclusion (C) between high-risk and low-risk groups. ∗p < 0.05, ∗∗p < 0.01, ∗∗∗p < 0.001.

**Fig. S11:**
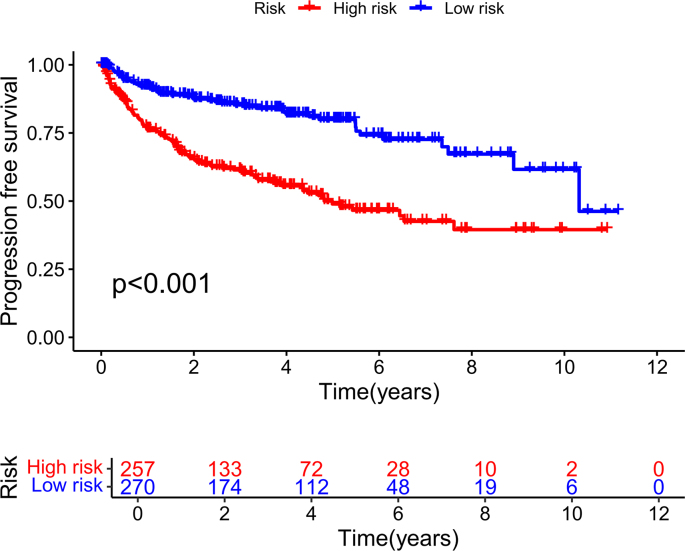
Progression-Free Survival in High-vs Low-Risk Groups.

HG with DRFs exhibit persistent T cell dysfunction and consistently high TIDE scores (Fig. S8). This may indicate that tumors in the HG are more likely to demonstrate immune evasion, making treatment more challenging. In the HG, the anti-tumor drugs to which tumors are sensitive are listed in Table S5. In LG, tumors exhibit sensitivity to a range of anti-tumor drugs that operate through diverse mechanisms of action, as detailed in Table S6. Anti-tumor drugs with no significant sensitivity differences between groups are presented in Table S7.

## 4 Discussion

Renal malignancies constitute a major global health concern, representing a leading contributor to cancer-related morbidity worldwide. Given its high mortality rate, it is crucial to continue exploring new treatment strategies for kidney cancer to improve patient outcomes and survival rates. Emerging research suggests that disulfidptosis and ferroptosis are crucial in the progression of tumors, influencing various aspects of tumor biology and development [4,5]. Despite their significance, no prognostic model incorporating these mechanisms has been established for KIRC. Our findings reveal that the nomogram derived from this model demonstrates robust predictive performance, while drug sensitivity analysis provides valuable insights for tailoring tumor treatments.

The disulfidptosis-related genes and ferroptosis-related genes selected for our model construction were derived from validated gene sets, which screened out genes associated with other forms of cell death. The resulting model demonstrated superior prognostic accuracy compared to traditional clinical staging systems. In our study, we employed a range of algorithms, including LASSO, to identify key biomarkers. The results of the model revealed that HG had lower survival rates than the LG. We developed a nomogram, which proved to be an effective and reliable tool for predicting patient survival outcomes. Ma X et al. created the prognostic model using disulfidptosis and ferroptosis-related genes, which was validated for its prognostic value in lung adenocarcinoma [6]. Similarly, Xu L et al. developed a prognostic model incorporating disulfidptosis and ferroptosis, which yielded promising results in breast cancer [18]. These findings, along with others, highlight the strong predictive potential of prognostic models based on disulfidptosis and ferroptosis, demonstrating their valuable role in cancer prognosis.

Among DRFs that we selected to build the prognostic model, EPAS1 expression is influenced by super-enhancers [19]. In breast cancer treatment, RRM2 is upregulated and contributes to resistance against GTI-2040, tamoxifen, Adriamycin, and cisplatin [20]. Kremer DM et al. have demonstrated that exogenous cysteine can selectively exploit GOT1-knockdown cells and tumors. This metabolic reprogramming can be leveraged through dietary means, which has significant implications for the nutrient landscape within pancreatic tumors [21]. Role of SLC40A1 in Iron Homeostasis and HCC Pathogenesis. As the primary cellular iron exporter, SLC40A1 critically regulates intracellular iron homeostasis, a process implicated in tumorigenesis across multiple cancer types. In hepatocellular carcinoma (HCC), iron overload is a recognized oncogenic driver: excess hepatic iron accumulation drives oxidative damage and genomic instability via iron-dependent enzyme dysregulation (e.g., mitochondrial respiration, lipid metabolism) [22]. This mechanistic link positions SLC40A1 as a potential modulator of HCC progression through iron efflux capacity. Our DRFs prognostic model demonstrated robust prognostic accuracy in patient cohorts, outperforming traditional clinicopathological metrics. This improvement underscores its translational potential for risk-stratified clinical management. The GSEA results can also provide clues about the mechanisms by which DRFs exert their effects on KIRC. The coagulation cascades may promote cancer growth and metastasis [23]. Recent evidence suggests that a hypercoagulable state is not merely a secondary effect caused by the presence of a tumor, but rather actively promotes the development and dissemination of the tumor. Epigenetic regulation angle Emerging evidence indicates that CYP1B1 overexpression is mechanistically involved in RCC tumorigenesis [24]. Analysis of the TME in KIRC patients demonstrated that infiltration of CD8^+^ T cells in high-risk samples correlates with poorer prognosis. This suggests that CD8^+^ T cells could serve as a promising target for immunotherapy, offering a potential therapeutic approach for improving patient outcomes. This finding offers valuable insights for identifying future immunotherapy targets in KIRC.

Drug sensitivity profiling pinpointed PI3K and cell cycle pathways as key vulnerability nodes, critical for regulating oncogenic proliferation-metabolism coupling. They represent potential targets for developing personalized treatment for KIRC. The mTOR pathway is often hyperactivated in cancer cells, making the inhibition of mTOR signaling a promising and effective approach for molecular targeted therapy in human cancers. The PI3K/mTOR and cell cycle pathways are crucial regulators of KIRC cell biology. Targeting these pathways could offer new opportunities for personalized treatment strategies.

Our study presents several notable strengths. First and foremost, we introduced a novel predictive model-a method that has not been reported before—which distinguishes our work from previous studies. For instance, while Feng X et al. constructed a robust model to forecast lymph node metastasis in kidney cancer [25], their study did not incorporate TMB analysis or investigate anticancer drug selection. In contrast, our research not only includes TMB analysis but also provides a detailed classification of anticancer drugs deemed critical for both HG and LG. Moreover, unlike the KIRC model presented by Li L et al. [26], we validated the key genes expression to construct our risk score via RT-qPCR-a step they did not undertake. Similarly, compared to the copper poisoning-related gene model from Mei W et al. [27], our risk score underwent more extensive validation using an external dataset, thereby yielding more reliable results. Moreover, our model demonstrated good predictive value, with an AUC of 0.765, surpassing the 0.750 AUC reported by Zhang L et al. [28]. However, we should acknowledge notable limitations in this study. While RT-PCR validation confirmed differential expression of signature genes between tumor and normal cell lines, further experimental validation of the model's biological mechanisms remains essential. Additionally, independent validation in prospective clinical cohorts is required to establish its clinical utility.

## 5 Conclusion

Overall, we combined the genes of dual-stream death and ferroptosis to construct a risk model and a nomogram. It has good predictive power and guidance for treatment. Worse outcomes in the HG may be linked to transmembrane transport functions and a higher TMB. However, due to various limitations, more clinical controlled studies are needed to clarify the model's value in drug sensitivity. Although clinical trials are planned to validate the model's predictive robustness, confirmatory bench experiments are critically needed to decipher its underlying biological mechanisms governing patient outcomes.

## Informed consent

For this type of study formal consent is not required.

## Ethics approval and consent

This article does not contain any studies with human participants or animals performed by any of the authors.

## Availability of data and material

Data is provided within the manuscript or supplementary information files.

## Funding

This study was Project supported by 10.13039/501100004479Jiangxi Provincial Natural Science Foundation (Grant number: 20212BAB206050), and Science and Technology Plan of Jiangxi Provincial Administration of Traditional Chinese Medicine (Grant number: 2022A360). Role of the Funding: The funding had no role in the design and conduct of the study; collection, management, analysis, and interpretation of the data; preparation, review, or approval of the manuscript; and decision to submit the manuscript for publication.

## Declaration of competing interest

The authors declare that they have no known competing financial interests or personal relationships that could have appeared to influence the work reported in this paper.

## Figures and Tables

**Fig. S9:**
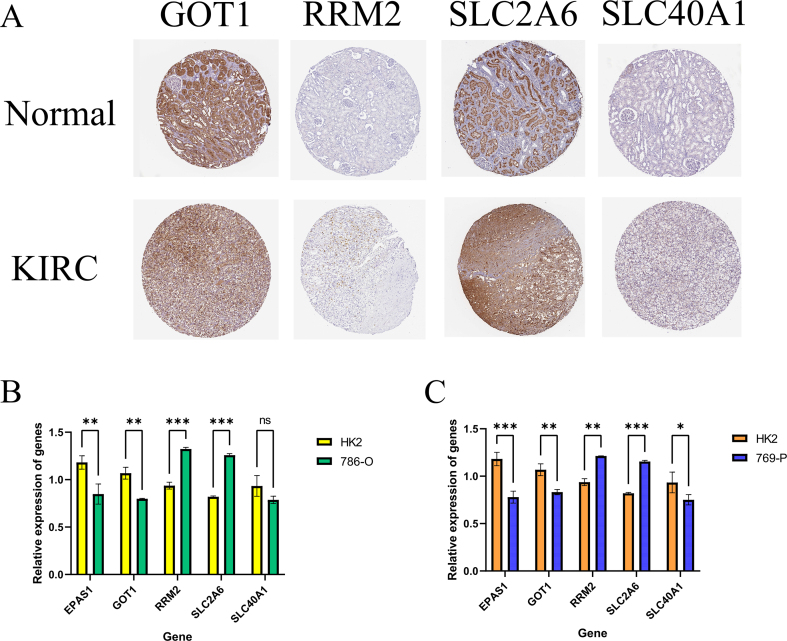
Immunohistochemical staining images of partial DRFs proteins in tumor cell lines and normal cell lines (A); Relative expression of 5 DRFs in tumor cell lines and normal cell lines (B, C). ∗p < 0.05, ∗∗p < 0.01, ∗∗∗p < 0.001, ∗∗∗∗p < 0.0001.

**Fig. S10:**
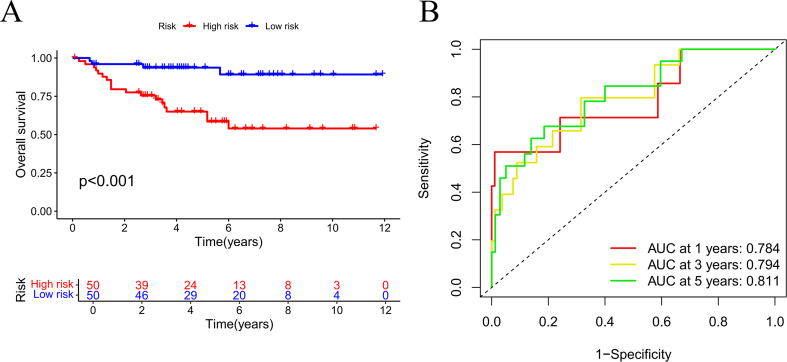
E-MTAB-1980 External validation. Survival analysis (A) and ROC curves (B) for the external validation set.

## Data Availability

Data is provided within the manuscript or supplementary information files.

## Abbreviations

ccRCC: Clear cell renal cell carcinoma; DCA: Decision curve analysis; DRFs: Disulfidptosis-related and ferroptosis-related genes; FDR: False discovery rate; GSEA: Gene set enrichment analysis; GSVA: Gene Set Variation Analysis; HG: high-risk group; HCC: Hepatocellular carcinoma; HPA: Human Protein Atlas; HR: Hazard ratios; IC50: Half maximal inhibitory concentration; KIRC: kidney renal clear cell carcinoma; K-M survival analysis: Kaplan-Meier survival analysis; LG: low-risk group; LASSO: Least absolute shrinkage and selection operator; NCCN: National Comprehensive Cancer Network; OS: Overall survival; PFS: Progression-Free Survival; PCA: Principal component analysis; PPI: Protein-protein interaction; ROC: Receiver operating characteristic; TAM: Tumor-associated macrophage; TCGA: The Cancer Genome Atlas; TIDE: Tumor immune dysfunction and exclusion; TIMER: Tumor Immune Estimation Resource; TMB: Tumor mutation burden; TME: Tumor microenvironment.
