# Supplementary material for: Integrated machine learning identifies disulfidptosis-related and ferroptosis-related genes to evaluate survival prognosis and treatment efficacy in kidney renal clear cell carcinoma
Source: Biochem Biophys Rep. 2025 Jul 12;43:102102. doi: 10.1016/j.bbrep.2025.102102 (PMC12280411; doi:10.1016/j.bbrep.2025.102102)
Supplement: Multimedia component 1 [file mmc1.docx]

**Table S1** Primer sequences for 5 DRFs.

| **Gene id** | **Primer F** | **Primer R** |
| --- | --- | --- |
| EPAS1 | CGGAGGTGTTCTATGAGCTGG | AGCTTGTGTGTTCGCAGGAA |
| GOT1 | ATGGCACCTCCGTCAGTCT | AGTCATCCGTGCGATATGCTC |
| RRM2 | CACGGAGCCGAAAACTAAAGC | TCTGCCTTCTTATACATCTGCCA |
| SLC2A6 | CCGGACTACGACACCTTCC | GGATGTGTAGACCAGGGCATA |
| SLC40A1 | CTACTTGGGGAGATCGGATGT | CTGGGCCACTTTAAGTCTAGC |

**Abbreviation:** DRFs: Disulfidptosis-related and ferroptosis-related genes.
