# Supplementary material for: Integrated machine learning identifies disulfidptosis-related and ferroptosis-related genes to evaluate survival prognosis and treatment efficacy in kidney renal clear cell carcinoma
Source: Biochem Biophys Rep. 2025 Jul 12;43:102102. doi: 10.1016/j.bbrep.2025.102102 (PMC12280411; doi:10.1016/j.bbrep.2025.102102)
Supplement: Multimedia component 2 [file mmc2.docx]

**Table S2** 52 DRFs identified through Univariate Cox Regression Analysis.

| **DRFs** | **HR** | **HR.95L** | **HR.95H** | **P-value** |
| --- | --- | --- | --- | --- |
| ACO1 | 0.557 | 0.414 | 0.751 | < 0.00001 |
| AGPAT3 | 0.68 | 0.518 | 0.894 | 0.006 |
| AURKA | 1.721 | 1.363 | 2.174 | < 0.00001 |
| BAP1 | 0.651 | 0.46 | 0.921 | 0.015 |
| BID | 2.111 | 1.348 | 3.306 | 0.001 |
| BNIP3 | 0.758 | 0.631 | 0.911 | 0.003 |
| CARS1 | 2.419 | 1.398 | 4.187 | 0.002 |
| CHMP5 | 0.571 | 0.434 | 0.75 | < 0.00001 |
| CHMP6 | 0.713 | 0.521 | 0.975 | 0.034 |
| DDIT3 | 1.304 | 1.012 | 1.68 | 0.04 |
| DPP4 | 0.787 | 0.698 | 0.888 | < 0.00001 |
| EGFR | 0.821 | 0.686 | 0.983 | 0.032 |
| EIF2S1 | 0.609 | 0.448 | 0.828 | 0.002 |
| EMC2 | 0.699 | 0.508 | 0.961 | 0.027 |
| EPAS1 | 0.701 | 0.607 | 0.81 | < 0.00001 |
| FANCD2 | 2.04 | 1.423 | 2.925 | < 0.00001 |
| G6PD | 1.925 | 1.455 | 2.547 | < 0.00001 |
| GABARAPL2 | 0.495 | 0.331 | 0.741 | 0.001 |
| GCLC | 0.571 | 0.427 | 0.763 | < 0.00001 |
| GOT1 | 0.568 | 0.452 | 0.713 | < 0.00001 |
| HERPUD1 | 0.519 | 0.384 | 0.702 | < 0.00001 |
| IL33 | 0.804 | 0.676 | 0.957 | 0.014 |
| LPCAT3 | 0.745 | 0.608 | 0.914 | 0.005 |
| LURAP1L | 0.685 | 0.562 | 0.836 | < 0.00001 |
| MAP3K5 | 0.645 | 0.499 | 0.834 | 0.001 |
| MAPK1 | 0.698 | 0.555 | 0.878 | 0.002 |
| MAPK3 | 0.609 | 0.451 | 0.821 | 0.001 |
| MAPK8 | 0.625 | 0.451 | 0.866 | 0.005 |
| MAPK9 | 0.632 | 0.452 | 0.884 | 0.007 |
| NCOA4 | 0.645 | 0.526 | 0.791 | < 0.00001 |
| NFE2L2 | 0.725 | 0.555 | 0.948 | 0.019 |
| NFS1 | 0.6 | 0.377 | 0.956 | 0.032 |
| NQO1 | 1.327 | 1.037 | 1.697 | 0.024 |
| NRAS | 0.746 | 0.584 | 0.952 | 0.018 |
| PEBP1 | 0.537 | 0.395 | 0.73 | < 0.00001 |
| PRKAA2 | 0.591 | 0.486 | 0.717 | < 0.00001 |
| RB1 | 0.785 | 0.632 | 0.975 | 0.029 |
| RRM2 | 1.652 | 1.343 | 2.032 | < 0.00001 |
| SCP2 | 0.66 | 0.53 | 0.822 | < 0.00001 |
| SIRT1 | 0.68 | 0.535 | 0.864 | 0.002 |
| SLC1A4 | 0.727 | 0.548 | 0.966 | 0.028 |
| SLC1A5 | 1.604 | 1.193 | 2.157 | 0.002 |
| SLC2A6 | 1.708 | 1.305 | 2.235 | < 0.00001 |
| SLC38A1 | 0.791 | 0.637 | 0.982 | 0.033 |
| SLC40A1 | 0.66 | 0.555 | 0.784 | < 0.00001 |
| SLC7A11 | 1.52 | 1.134 | 2.038 | 0.005 |
| SNX4 | 0.662 | 0.513 | 0.855 | 0.002 |
| SRC | 1.517 | 1.089 | 2.114 | 0.014 |
| TLR4 | 0.702 | 0.558 | 0.885 | 0.003 |
| TXNIP | 0.761 | 0.615 | 0.941 | 0.012 |
| VDAC2 | 0.656 | 0.465 | 0.925 | 0.016 |
| ZEB1 | 0.775 | 0.63 | 0.952 | 0.015 |

**Abbreviation:** DRFs: Disulfidptosis-related and ferroptosis-related genes.
