# Supplementary material for: Integrated machine learning identifies disulfidptosis-related and ferroptosis-related genes to evaluate survival prognosis and treatment efficacy in kidney renal clear cell carcinoma
Source: Biochem Biophys Rep. 2025 Jul 12;43:102102. doi: 10.1016/j.bbrep.2025.102102 (PMC12280411; doi:10.1016/j.bbrep.2025.102102)
Supplement: Multimedia component 3 [file mmc3.docx]

**Table S3** 5 DRFs identified through multivariate Cox analysis.

| **Genes** | **Coef** |
| --- | --- |
| EPAS1 | -0.25134 |
| GOT1 | -0.19648 |
| RRM2 | 0.327005 |
| SLC2A6 | 0.248502 |
| SLC40A1 | -0.19625 |

Abbreviation: DRFs: Disulfidptosis-related and ferroptosis-related genes.
