# Supplementary material for: Integrated machine learning identifies disulfidptosis-related and ferroptosis-related genes to evaluate survival prognosis and treatment efficacy in kidney renal clear cell carcinoma
Source: Biochem Biophys Rep. 2025 Jul 12;43:102102. doi: 10.1016/j.bbrep.2025.102102 (PMC12280411; doi:10.1016/j.bbrep.2025.102102)
Supplement: Multimedia component 4 [file mmc4.docx]

**Table S4** GSEA pathways for different risk groups.

| **Pathways** | **Group** |
| --- | --- |
| **c2.cp.kegg.Hs.symbols.gmt** |  |
| KEGG_ENDOCYTOSIS | Low risk |
| KEGG_ENDOMETRIAL_CANCER | Low risk |
| KEGG_NEUROTROPHIN_SIGNALING_PATHWAY | Low risk |
| KEGG_VALINE_LEUCINE_AND_ISOLEUCINE_DEGRADATION | Low risk |
| KEGG_VASCULAR_SMOOTH_MUSCLE_CONTRACTION | Low risk |
| KEGG_COMPLEMENT_AND_COAGULATION_CASCADES | High risk |
| KEGG_DRUG_METABOLISM_CYTOCHROME_P450 | High risk |
| KEGG_DRUG_METABOLISM_OTHER_ENZYMES | High risk |
| KEGG_METABOLISM_OF_XENOBIOTICS_BY_CYTOCHROME_P450 | High risk |
| KEGG_RETINOL_METABOLISM | High risk |
| **c5.go.Hs.symbols.gmt** |  |
| GOBP_HUMORAL_IMMUNE_RESPONSE | High risk |
| GOCC_IMMUNOGLOBULIN_COMPLEX | High risk |
| GOCC_IMMUNOGLOBULIN_COMPLEX_CIRCULATING | High risk |
| GOMF_ANTIGEN_BINDING | High risk |
| GOMF_IMMUNOGLOBULIN_RECEPTOR_BINDING | High risk |
| GOBP_INORGANIC_ANION_TRANSMEMBRANE_TRANSPORT | Low risk |
| GOBP_INORGANIC_ANION_TRANSPORT | Low risk |
| GOBP_SPROUTING_ANGIOGENESIS | Low risk |
| GOCC_APICAL_PART_OF_CELL | Low risk |
| GOMF_INORGANIC_ANION_TRANSMEMBRANE_TRANSPORTER_ACTIVITY | Low risk |

**Abbreviation:** GSEA: Gene Set Enrichment Analysis.
