# Supplementary material for: Integrated machine learning identifies disulfidptosis-related and ferroptosis-related genes to evaluate survival prognosis and treatment efficacy in kidney renal clear cell carcinoma
Source: Biochem Biophys Rep. 2025 Jul 12;43:102102. doi: 10.1016/j.bbrep.2025.102102 (PMC12280411; doi:10.1016/j.bbrep.2025.102102)
Supplement: Multimedia component 5 [file mmc5.docx]

**Table S5** Antineoplastic drug sensitivity (sensitive group: high).

| **Drugs** | **Low-risk group** |  | **High-risk group** | **P-value** |
| --- | --- | --- | --- | --- |
|  | **IC50 (25%-75%）** |  | **IC50 (25%-75%）** |  |
| **Chromatin other** | | | | |
| Vinblastine_1004 | 0.027(0.014-0.044) |  | 0.021(0.01-0.042) | 0.015 |
| Vinorelbine_2048 | 0.047(0.026-0.09) |  | 0.039(0.015-0.104) | 0.028 |
| VE821_2111 | 59.654(46.521-82.685) |  | 56.424(37.623-79.149) | 0.013 |
| AZD6738_1917 | 7.649(5.828-10.278) |  | 6.976(4.825-10.512) | 0.008 |
| **RTK signaling** | | | | |
| AZD2014_1441 | 8.886(6.997-11.617) |  | 6.98(5.278-9.025) | 0.01 |
| **Cell cycle** | | | | |
| Palbociclib_1054 | 40.653(28.944-57.117) |  | 34.305(22.049-52.996) | 0.022 |
| PD0325901_1060 | 1.618(1.267-2.401) |  | 1.45(1.05-2.226) | 0.043 |
| Topotecan_1808 | 1.252(0.738-1.889) |  | 0.92(0.523-1.752) | 0.014 |
| Podophyllotoxin.bromide_1825 | 0.474(0.351-0.681) |  | 0.443(0.302-0.776) | 0.007 |
| Savolitinib_1936 | 13.579(11.307-16.8) |  | 13.225(10.638-16.189) | 0.02 |
| **Chromatin other** | | | | |
| Irinotecan_1088 | 13.507(8.752-23.072) |  | 10.505(5.847-21.678) | 0.003 |
| Luminespib_1559 | 0.105(0.064-0.161) |  | 0.1(0.048-0.183) | 0.028 |
| Leflunomide_1578 | 143.11(120.487-174.515) |  | 139.945(112.784-178.148) | 0.002 |
| MIRA.1_1931 | 230.71(172.586-291.787) |  | 225.986(163.207-304.526) | 0.018 |
| **IGF1R signaling** | | | | |
| Oxaliplatin_1089 | 42.635(28.907-60.587) |  | 40.086(23.206-68.76) | 0.002 |
| Oxaliplatin_1806 | 156.197(118.433-206.854) |  | 128.287(89.763-191.434) | 0.028 |
| **p53 pathway** | | | | |
| Niraparib_1177 | 71.978(50.082-105.764) |  | 65.171(43.2-111.646) | 0.007 |
| Mitoxantrone_1810 | 1.717(1.119-2.629) |  | 1.632(0.942-3.352) | 0.007 |
| **PI3K/MTOR signaling** | | | | |
| Camptothecin_1003 | 0.09(0.055-0.143) |  | 0.077(0.042-0.152) | 0.004 |
| Cisplatin_1005 | 27.1(15.748-46.119) |  | 20.325(10.036-47.555) | 0.017 |
| Gemcitabine_1190 | 0.522(0.293-1.081) |  | 0.391(0.186-0.86) | 0.003 |
| GSK269962A_1192 | 18.457(16.097-21.005) |  | 18.395(15.149-21.226) | 0.035 |
| EPZ5676_1563 | 239.907(201.149-296.272) |  | 237.15(188.392-323.438) | 0.04 |
| Dactinomycin_1911 | 0.008(0.005-0.012) |  | 0.007(0.005-0.012) | 0.002 |

**Abbreviation:** IC50: Half maximal inhibitory concentration.
