# Supplementary material for: Integrated machine learning identifies disulfidptosis-related and ferroptosis-related genes to evaluate survival prognosis and treatment efficacy in kidney renal clear cell carcinoma
Source: Biochem Biophys Rep. 2025 Jul 12;43:102102. doi: 10.1016/j.bbrep.2025.102102 (PMC12280411; doi:10.1016/j.bbrep.2025.102102)
Supplement: Multimedia component 6 [file mmc6.docx]

**Table S6** Antineoplastic drug sensitivity (sensitive group: low).

| **Drugs** | **Low-risk group** |  | **High-risk group** | **P-value** |
| --- | --- | --- | --- | --- |
|  | **IC50 (25%-75%）** |  | **IC50 (25%-75%）** |  |
| **Chromatin other** | | | | |
| Wee1.Inhibitor_1046 | 6.493(4.613-9.596) |  | 6.832(4.814-11.965) | 0.011 |
| ZM447439_1050 | 18.524(15.389-21.902) |  | 19.558(15.617-23.71) | 0.006 |
| YK.4.279_1239 | 8.948(5.338-14.338) |  | 9.291(4.597-19.912) | 0.011 |
| Wnt.C59_1622 | 60.736(51.103-71.356) |  | 72.287(56.539-94.924) | 0.002 |
| VSP34_8731_1734 | 9.586(6.693-13.831) |  | 9.905(6.715-16.063) | 0.024 |
| Zoledronate_1802 | 38.09(30.056-49.458) |  | 44.626(34.71-61.159) | 0.005 |
| Vincristine_1818 | 0.121(0.072-0.213) |  | 0.156(0.078-0.418) | < 0.00001 |
| **ERK MAPK signaling** | | | | |
| Ulixertinib_1908 | 14.697(11.009-19.018) |  | 17.372(13.291-25.438) | 0.004 |
| UMI.77_1939 | 12.206(8.62-17.249) |  | 15.975(11.28-28.375) | 0.037 |
| Ulixertinib_2047 | 7.677(6.046-10.347) |  | 10.22(7.372-13.77) | 0.001 |
| **Other, kinases** | | | | |
| AZD5438_1401 | 7.565(5.469-10.556) |  | 7.984(5.102-15.921) | 0.007 |
| AZD5582_1617 | 7.857(4.922-12.672) |  | 8.169(4.575-17.483) | 0.002 |
| AZD5991_1720 | 63.452(39.953-103.76) |  | 70.598(36.708-148.611) | 0.015 |
| **Cell cycle** | | | | |
| PD173074_1049 | 48.494(36.782-65.896) |  | 64.748(44.266-91.908) | 0.007 |
| RO.3306_1052 | 13.941(11.171-16.83) |  | 17.791(13.851-22.055) | 0.024 |
| Paclitaxel_1080 | 0.054(0.03-0.1) |  | 0.065(0.03-0.141) | 0.003 |
| Rapamycin_1084 | 0.109(0.071-0.147) |  | 0.125(0.074-0.196) | 0.002 |
| Tozasertib_1096 | 16.003(12.234-20.565) |  | 19.932(14.486-26.642) | 0.001 |
| Talazoparib_1259 | 22.565(13.066-38.029) |  | 22.83(10.131-49.974) | 0.005 |
| Temozolomide_1375 | 369.34(285.291-528.376) |  | 377.377(273.938-532.688) | 0.015 |
| Ruxolitinib_1507 | 113.357(92.181-138.311) |  | 139.041(104.68-183.256) | 0.002 |
| Pevonedistat_1529 | 1.607(0.917-2.581) |  | 2.173(1.128-4.941) | 0.006 |
| PFI3_1620 | 176.65(152.762-207.546) |  | 190.035(160.298-236.118) | 0.012 |
| PCI.34051_1621 | 77.374(60.191-101.026) |  | 87.995(58.277-136.926) | 0.028 |
| RVX.208_1625 | 110.153(93.763-127.03) |  | 121.882(94.942-150.887) | 0.013 |
| PAK_5339_1730 | 10.33(8.87-12.277) |  | 10.995(9.168-13.069) | 0.005 |
| Teniposide_1809 | 1.431(0.79-2.617) |  | 1.579(0.722-3.84) | 0.001 |
| Sinularin_1838 | 28.85(20.991-39.759) |  | 37.483(25.178-62.947) | 0.013 |
| Telomerase.Inhibitor.IX_1930 | 1.453(1.077-2.055) |  | 1.706(1.103-2.627) | 0.003 |
| P22077_1933 | 63.095(47.826-90.626) |  | 109.803(72.806-167.178) | < 0.00001 |
| Sepantronium.bromide_1941 | 0.01(0.007-0.015) |  | 0.017(0.01-0.031) | 0.001 |
| **Chromatin other** |  |  |  |  |
| LCL161_1557 | 117.824(92.952-151.012) |  | 157.597(122.794-210.849) | 0.009 |
| IWP.2_1576 | 15.196(12.728-17.892) |  | 16.221(13.132-19.676) | 0.007 |
| LGK974_1598 | 50.098(40.132-65.941) |  | 57.418(42.578-81.407) | 0.012 |
| LY2109761_1852 | 131.944(97.197-185.128) |  | 190.534(128.753-308.546) | 0.025 |
| KRAS..G12C..Inhibitor.12_1855 | 62.853(44.266-90.197) |  | 84.676(52.205-141.383) | 0.004 |
| MIM1_1996 | 48.109(35.957-61.356) |  | 50.561(34.623-68.884) | 0.001 |
| **EGFR signaling** | | | | |
| AZD4547_1786 | 15.676(12.626-19.593) |  | 19.513(14.939-27.444) | 0.004 |
| **IGF1R signaling** | | | | |
| Olaparib_1017 | 67.402(41.436-95.983) |  | 81.495(47.256-134.054) | 0.003 |
| OTX015_1626 | 11.011(7.541-15.704) |  | 12.569(7.475-20.568) | 0.044 |
| OF.1_1853 | 47.205(37.238-61.297) |  | 72.52(53.892-112.168) | 0.048 |
| Osimertinib_1919 | 5.057(4.028-6.418) |  | 6.543(4.784-8.745) | 0.01 |
| **p53 pathway** | | | | |
| MK.1775_1179 | 1.557(1.172-2.222) |  | 1.764(1.254-2.865) | < 0.00001 |
| ML323_1629 | 75.976(64.419-90.846) |  | 97.131(75.403-121.437) | < 0.00001 |
| NVP.ADW742_1932 | 13.495(10.219-18.139) |  | 16.563(13.12-21.825) | 0.036 |
| **PI3K/MTOR signaling** | | | | |
| Cytarabine_1006 | 5.073(3.257-8.862) |  | 5.075(2.887-10.513) | < 0.00001 |
| Docetaxel_1007 | 0.01(0.008-0.013) |  | 0.01(0.007-0.015) | 0.002 |
| Crizotinib_1083 | 23.822(18.227-31.679) |  | 23.832(17.393-33) | 0.001 |
| GSK1904529A_1093 | 63.199(47.836-82.841) |  | 83.143(56.762-122.318) | 0.021 |
| Dinaciclib_1180 | 0.045(0.035-0.068) |  | 0.066(0.044-0.117) | < 0.00001 |
| Bortezomib_1191 | 0.007(0.006-0.009) |  | 0.008(0.006-0.009) | 0.012 |
| Daporinad_1248 | 0.007(0.005-0.013) |  | 0.015(0.009-0.03) | < 0.00001 |
| BMS.345541_1249 | 20.533(15.613-29.342) |  | 33.261(22.282-55.643) | < 0.00001 |
| IAP_5620_1428 | 137.371(105.222-189.34) |  | 171.08(116.317-265.407) | 0.003 |
| Epirubicin_1511 | 0.334(0.218-0.529) |  | 0.359(0.191-0.633) | 0.001 |
| Cyclophosphamide_1512 | 163.315(136.356-205.081) |  | 171.026(133.89-225.104) | 0.029 |
| GSK2606414_1618 | 37.596(30.083-46.863) |  | 45.212(32.767-62.188) | 0.016 |
| I.BET.762_1624 | 23.722(17.834-32.844) |  | 31.767(18.722-47.559) | 0.011 |
| Entospletinib_1630 | 38.769(31.186-50.709) |  | 40.926(30.178-53.787) | 0.012 |
| CDK9_5576_1708 | 0.527(0.379-0.77) |  | 0.626(0.413-1.378) | 0.006 |
| CDK9_5038_1709 | 0.079(0.051-0.129) |  | 0.084(0.05-0.209) | 0.039 |
| Eg5_9814_1712 | 0.039(0.023-0.061) |  | 0.041(0.022-0.096) | 0.041 |
| ERK_2440_1713 | 12.62(8.238-17.98) |  | 13.359(7.616-26.461) | 0.022 |
| Ibrutinib_1799 | 71.485(54.318-98.804) |  | 109.589(76.467-159.771) | 0.001 |
| Carmustine_1807 | 379.734(313.059-488.515) |  | 512.313(385.176-666.099) | < 0.00001 |
| Dactinomycin_1811 | 0.079(0.056-0.107) |  | 0.084(0.056-0.141) | < 0.00001 |
| Fulvestrant_1816 | 84.564(71.159-111.191) |  | 89.191(66.23-133.912) | 0.021 |
| Docetaxel_1819 | 0.057(0.029-0.13) |  | 0.108(0.045-0.382) | < 0.00001 |
| Dihydrorotenone_1827 | 2.054(1.568-2.569) |  | 2.961(2.159-3.941) | 0.022 |
| Gallibiscoquinazole_1830 | 11.378(9.505-14.215) |  | 14.436(11.044-20.004) | < 0.00001 |
| Elephantin_1835 | 27.796(19.173-39.458) |  | 28.765(14.759-54.015) | 0.013 |
| BDP.00009066_1866 | 10.112(8.195-12.451) |  | 10.896(7.85-14.095) | < 0.00001 |
| Cediranib_1922 | 7.269(5.943-8.641) |  | 9.757(7.315-12.892) | 0.017 |
| GDC0810_1925 | 131.309(113.503-158.226) |  | 137.267(112.999-175.56) | 0.002 |
| I.BRD9_1928 | 74.163(55.021-91.268) |  | 82.334(61.617-116.486) | < 0.00001 |
| BPD.00008900_1998 | 89.125(72.486-114.266) |  | 90.24(62.087-125.991) | 0.005 |
| BIBR.1532_2043 | 122.013(94.833-165.032) |  | 144.215(106.771-218.499) | 0.018 |
| GSK591_2110 | 88.655(69.881-125.545) |  | 97.877(68.005-133.599) | 0.007 |
| BMS.754807_2171 | 1.328(0.934-1.718) |  | 1.478(0.928-2.133) | 0.004 |

**Abbreviation:** IC50: Half maximal inhibitory concentration.
