# Supplementary material for: Integrated machine learning identifies disulfidptosis-related and ferroptosis-related genes to evaluate survival prognosis and treatment efficacy in kidney renal clear cell carcinoma
Source: Biochem Biophys Rep. 2025 Jul 12;43:102102. doi: 10.1016/j.bbrep.2025.102102 (PMC12280411; doi:10.1016/j.bbrep.2025.102102)
Supplement: Multimedia component 7 [file mmc7.docx]

**Table S7** Antineoplastic drug sensitivity (no obviously sensitive group).

| **Drugs** | **Low-risk group** |  | **High-risk group** | **P-value** |
| --- | --- | --- | --- | --- |
|  | **IC50 (25%-75%）** |  | **IC50 (25%-75%）** |  |
| **Chromatin other** | | | | |
| Vorinostat_1012 | 4.417(3.265-6.306) |  | 3.924(2.712-5.905) | 0.457 |
| X5.Fluorouracil_1073 | 120.781(75.688-226.392) |  | 90.445(40.744-189.802) | 0.051 |
| XAV939_1268 | 91.314(78.57-107.269) |  | 68.86(57.758-85.091) | 0.518 |
| Uprosertib_1553 | 20.425(15.254-26.894) |  | 17.432(12.553-26.474) | 0.224 |
| VE.822_1613 | 29.063(21.386-39.361) |  | 25.81(17.965-35.221) | 0.101 |
| WZ4003_1614 | 44.655(33.941-54.618) |  | 36.814(28.679-49.773) | 0.995 |
| Venetoclax_1909 | 8.849(6.994-11.097) |  | 7.428(5.476-11.27) | 0.608 |
| WIKI4_1940 | 42.696(38.329-47.947) |  | 37.598(32.744-44.235) | 0.392 |
| WEHI.539_1997 | 33.692(24.979-47.33) |  | 32.69(18.139-51.166) | 0.553 |
| VX.11e_2096 | 18.049(11.821-24.569) |  | 18.115(11.746-25.12) | 0.143 |
| KU.55933_1030 | 88.917(83.874-93.963) |  | 86.894(80.602-93.545) | 0.36 |
| Linsitinib_1510 | 41.464(31.931-55.297) |  | 41.605(30.828-59.181) | 0.376 |
| Lapatinib_1558 | 22.513(17.624-28.263) |  | 17.319(13.365-23.284) | 0.854 |
| IRAK4_4710_1716 | 132.018(105.427-161.962) |  | 133.486(102.321-175.15) | 0.115 |
| JAK1_8709_1718 | 64.144(50.618-80.039) |  | 59.528(44.658-83.983) | 0.599 |
| JAK_8517_1739 | 19.753(12.919-28.503) |  | 19.043(10.901-33.293) | 0.28 |
| MG.132_1862 | 0.201(0.176-0.232) |  | 0.193(0.163-0.234) | 0.185 |
| Ipatasertib_1924 | 38.489(27.804-48.11) |  | 29.323(21.13-41.421) | 0.547 |
| LJI308_2107 | 169.069(138.561-217.264) |  | 143.186(109.585-194.848) | 0.193 |
| JQ1_2172 | 9.21(6.688-12.794) |  | 10.826(7.477-15.801) | 0.076 |
| Uprosertib_2106 | 19.041(12.859-26.881) |  | 12.804(8.436-19.314) | 0.411 |
| **ERK MAPK signaling** | | | | |
| ULK1_4989_1733 | 11.24(7.51-17.087) |  | 8.272(5.236-13.007) | 0.57 |
| **Genome integrity** | | | | |
| AZD7762_1022 | 1.311(0.959-1.939) |  | 0.793(0.534-1.245) | 0.558 |
| AZD8055_1059 | 0.839(0.79-0.892) |  | 0.809(0.733-0.87) | 0.547 |
| **Other, kinases** | | | | |
| AZD5363_1916 | 20.606(16.19-26.846) |  | 17.106(12.185-22.764) | 0.819 |
| AZD6482_2169 | 23.508(18.704-29.183) |  | 21.419(15.988-28.416) | 0.582 |
| **RTK signaling** | | | | |
| AZD3759_1915 | 14.383(12.106-17.284) |  | 13.786(11.041-18.995) | 0.069 |
| **Cell cycle** | | | | |
| SB216763_1025 | 164.424(137.667-203.043) |  | 176.758(140.416-241.05) | 0.076 |
| Staurosporine_1034 | 0.047(0.028-0.076) |  | 0.048(0.025-0.083) | 0.058 |
| PLX.4720_1036 | 87.064(63.846-113.743) |  | 78.876(59.487-104.068) | 0.053 |
| Pictilisib_1058 | 4.555(3.297-6.534) |  | 3.485(2.484-4.905) | 0.957 |
| Sorafenib_1085 | 13.401(10.078-19.913) |  | 13.467(8.662-20.884) | 0.149 |
| PF.4708671_1129 | 51.522(46.75-58.015) |  | 45.367(38.532-53.415) | 0.536 |
| PRIMA.1MET_1131 | 101.668(71.382-150.998) |  | 76.771(44.785-147.655) | 0.135 |
| SB505124_1194 | 8.791(8.019-9.861) |  | 9.631(8.691-11.299) | 0.568 |
| Tamoxifen_1199 | 34.927(29.646-41.836) |  | 34.238(27.92-42.472) | 0.168 |
| Trametinib_1372 | 1.836(1.13-2.935) |  | 1.971(0.975-2.908) | 0.497 |
| Sapitinib_1549 | 52.149(42.514-65.262) |  | 52.251(39.494-68.377) | 0.277 |
| Taselisib_1561 | 9.371(5.652-13.294) |  | 7.351(4.102-12.296) | 0.652 |
| SCH772984_1564 | 13.706(9.378-20.495) |  | 14.107(8.297-21.418) | 0.089 |
| PRT062607_1631 | 28.442(23.45-35.921) |  | 22.151(17.41-27.774) | 0.361 |
| Ribociclib_1632 | 47.195(44.715-50.225) |  | 46.933(41.774-51.035) | 0.288 |
| Picolinici.acid_1635 | 160.053(141.592-191.705) |  | 160.631(128.344-209.804) | 0.092 |
| TAF1_5496_1732 | 42.902(29.995-57.709) |  | 51.018(34.025-78.279) | 0.349 |
| Selumetinib_1736 | 73.727(48.15-109.508) |  | 54.401(29.654-86.338) | 0.797 |
| Sabutoclax_1849 | 0.676(0.474-0.966) |  | 0.593(0.41-0.918) | 0.067 |
| Pyridostatin_2044 | 29.22(21.746-39.443) |  | 26.439(18.673-35.439) | 0.142 |
| **EGFR signaling** | | | | |
| AZD5153_1706 | 5.131(3.655-6.995) |  | 5.81(3.65-8.479) | 0.051 |
| **IGF1R signaling** | | | | |
| Obatoclax.Mesylate_1068 | 3.913(3.073-5.112) |  | 3.76(2.901-5.272) | 0.234 |
| OSI.027_1594 | 102.524(95.9-108.086) |  | 108.649(100.188-116.071) | 0.094 |
| **Other** | | | | |
| AZ960_1250 | 8.504(6.251-12.339) |  | 6.491(4.226-9.447) | 0.101 |
| AZD1208_1449 | 200.931(152.775-254.107) |  | 189.709(128.61-285.238) | 0.694 |
| AZD1332_1463 | 45.51(34.964-57.633) |  | 47.589(34.914-65.552) | 0.108 |
| **p53 pathway** | | | | |
| Navitoclax_1011 | 8.682(4.376-16.588) |  | 5.457(1.618-15.192) | 0.905 |
| Nilotinib_1013 | 35.148(25.479-51.136) |  | 34.693(23.205-55.882) | 0.512 |
| NU7441_1038 | 12.275(10.501-14.194) |  | 13.983(11.259-16.726) | 0.107 |
| Nutlin.3a...._1047 | 116.075(80.07-199.614) |  | 82.011(48.834-147.516) | 0.854 |
| Mirin_1048 | 118.24(88.112-159.333) |  | 97.077(67.087-145.285) | 0.381 |
| MK.2206_1053 | 22.044(17.519-27.433) |  | 16.871(13.179-23.197) | 0.437 |
| Nelarabine_1814 | 369.121(250.936-525.361) |  | 395.409(248.948-652.854) | 0.083 |
| MN.64_1854 | 104.715(79.724-131.532) |  | 109.694(82.508-166.672) | 0.052 |
| MK.8776_2046 | 27.332(21.811-36.892) |  | 19.889(13.343-28.969) | 0.844 |
| **PI3K/MTOR signaling** | | | | |
| Gefitinib_1010 | 26.39(21.305-31.484) |  | 24.48(18.334-32.867) | 0.318 |
| Doramapimod_1042 | 88.971(81.601-99.335) |  | 92.84(79.721-106.181) | 0.746 |
| Dactolisib_1057 | 0.185(0.133-0.286) |  | 0.185(0.121-0.334) | 0.108 |
| Dasatinib_1079 | 5.935(3.267-9.613) |  | 5.303(2.194-9.35) | 0.438 |
| BI.2536_1086 | 1.052(0.76-1.547) |  | 1.452(1.014-2.094) | 0.155 |
| BMS.536924_1091 | 8.36(6.625-10.727) |  | 7.993(5.591-11.019) | 0.197 |
| Erlotinib_1168 | 13.134(11.307-16.26) |  | 13.21(10.076-17.842) | 0.959 |
| Fulvestrant_1200 | 18.151(15.728-23.046) |  | 16.907(14.582-22.19) | 0.079 |
| EPZ004777_1237 | 172.347(130.551-235.668) |  | 154.447(112.348-236.1) | 0.272 |
| Dabrafenib_1373 | 114.159(86.932-146.052) |  | 80.901(58.592-115.18) | 0.751 |
| Entinostat_1593 | 10.92(7.59-15.804) |  | 7.243(4.251-12.382) | 0.805 |
| CZC24832_1615 | 159.527(139.897-191.198) |  | 145.213(121.122-179.128) | 0.117 |
| GSK343_1627 | 16.581(13.775-19.769) |  | 15.298(12.173-19.846) | 0.202 |
| ERK_6604_1714 | 30.29(29.644-30.981) |  | 30.149(29.305-30.869) | 0.198 |
| IGF1R_3801_1738 | 4.856(3.276-7.171) |  | 5.111(3.156-8.183) | 0.073 |
| Fludarabine_1813 | 159.865(100.09-256.251) |  | 126.567(70.084-235.953) | 0.225 |
| Buparlisib_1873 | 2.569(2.167-3.122) |  | 2.452(1.971-3.151) | 0.052 |
| AZD8186_1918 | 26.981(22.024-33.613) |  | 23.895(18.841-31.875) | 0.707 |
| GNE.317_1926 | 1.8(1.514-2.142) |  | 1.587(1.261-2.003) | 0.949 |
| GSK2578215A_1927 | 145.092(126.3-166.433) |  | 118.777(99.317-144.388) | 0.938 |
| Foretinib_2040 | 2.429(2.032-3.18) |  | 2.573(1.948-3.504) | 0.092 |

**Abbreviation:** IC50: Half maximal inhibitory concentration.
